# An Integrative Computational Framework Based on a Two-Step Random Forest Algorithm Improves Prediction of Zinc-Binding Sites in Proteins

**DOI:** 10.1371/journal.pone.0049716

**Published:** 2012-11-14

**Authors:** Cheng Zheng, Mingjun Wang, Kazuhiro Takemoto, Tatsuya Akutsu, Ziding Zhang, Jiangning Song

**Affiliations:** 1 National Engineering Laboratory for Industrial Enzymes and Key Laboratory of Systems Microbial Biotechnology, Tianjin Institute of Industrial Biotechnology, Chinese Academy of Sciences, Tianjin, China; 2 Department of Bioscience and Bioinformatics, Kyushu Institute of Technology, Iizuka, Fukuoka, Japan; 3 Bioinformatics Center, Institute for Chemical Research, Kyoto University, Uji, Kyoto, Japan; 4 State Key Laboratory of Agrobiotechnology, College of Biological Sciences, China Agricultural University, Beijing, China; 5 Department of Biochemistry and Molecular Biology, Faculty of Medicine, Monash University, Melbourne, Victoria, Australia; Koc University, Turkey

## Abstract

Zinc-binding proteins are the most abundant metalloproteins in the Protein Data Bank where the zinc ions usually have catalytic, regulatory or structural roles critical for the function of the protein. Accurate prediction of zinc-binding sites is not only useful for the inference of protein function but also important for the prediction of 3D structure. Here, we present a new integrative framework that combines multiple sequence and structural properties and graph-theoretic network features, followed by an efficient feature selection to improve prediction of zinc-binding sites. We investigate what information can be retrieved from the sequence, structure and network levels that is relevant to zinc-binding site prediction. We perform a two-step feature selection using random forest to remove redundant features and quantify the relative importance of the retrieved features. Benchmarking on a high-quality structural dataset containing 1,103 protein chains and 484 zinc-binding residues, our method achieved >80% recall at a precision of 75% for the zinc-binding residues Cys, His, Glu and Asp on 5-fold cross-validation tests, which is a 10%-28% higher recall at the 75% equal precision compared to SitePredict and zincfinder at residue level using the same dataset. The independent test also indicates that our method has achieved recall of 0.790 and 0.759 at residue and protein levels, respectively, which is a performance better than the other two methods. Moreover, AUC (the Area Under the Curve) and AURPC (the Area Under the Recall-Precision Curve) by our method are also respectively better than those of the other two methods. Our method can not only be applied to large-scale identification of zinc-binding sites when structural information of the target is available, but also give valuable insights into important features arising from different levels that collectively characterize the zinc-binding sites. The scripts and datasets are available at http://protein.cau.edu.cn/zincidentifier/.

## Introduction

Metalloproteins are proteins that can bind metal ions in order to fulfill the catalytic or structural requirement. Approximately one-quarter to one-third of all proteins require metal ions to carry out their functions [1], [2]. Zinc is the most abundant metal ion in the Protein Data Bank (PDB) database [3], [4], playing a wide range of functional roles in eukaryotic organisms [5]. It is estimated that about 40% of the zinc-binding proteins in the human proteome are transcription factors; the remaining 60% are primarily enzymes as well as other proteins including those involved in ion transport [6]. Due to the important structural and functional roles, the presence and location of the zinc-binding sites can provide important clues regarding the function of the protein [7]. Therefore, accurate prediction of zinc-binding sites is useful not only for the inference of protein function but also for the prediction of 3D structures of proteins that contain zinc ions, thereby providing an important assistance in facilitating protein functional annotation efforts.

A plethora of computational approaches have been proposed to address the task of predicting metal (e.g. zinc) binding sites. Many research groups attempt to accomplish this by using complex machine learning methods to train prediction models based on the primary sequences of the target proteins. For example, Lin *et al.* [8] previously employed a neural network approach to predict the metal binding residues based on the biochemical and biophysical features derived from sequence. In another study, Lin *et al.* [9] developed a support vector machine (SVM)-based method with improved classification performance that used sequence-derived physicochemical properties to predict 10 metal-binding classes and identify all metal binding proteins. Their results showed that several residue properties play more prominent roles than other properties for the prediction of the zinc binding sites, including hydrophobicity, solvent accessibility, polarity and composition. Menchetti *et al.* [10] trained SVM classifiers to identify all Cys and His (CHs) that coordinate with zinc in the target proteins. More specifically, they described the zinc-binding site using the sequence motif [CH] x(0–7) [CH] (x(0–7) denotes a subsequence with a length from 0 to 7 residues) which underlies the correlation between the nearby residues. Their method achieved 60% precision at 60% recall for predicting zinc-binding Cys and His based on 5-fold cross-validation tests. In a follow-up study by the same group [11], the sequence motif of zinc-binding sites was integrated into two predictors to predict the zinc-binding sites and annotate metal-binding site proteins at the proteome level. Their method achieved an AUC (area under the ROC curve) of 0.890 and an AURPC (area under the Recall-Precision curve) of 0.500 for predicting zinc-binding sites using 5-fold cross-validation tests based on 2,428 protein chains. Shu *et al.* [12] recently combined SVM-based and homology-based predictions to identify zinc-binding sites by focusing on four residue types- Cys, His, Glu and Asp (CHED). Their results showed that CHs could be more readily predicted compared to EDs. Lippi *et al.* [13] developed a web server to predict the metal-binding sites and disulfide bridges from sequence. Andreini *et al.* [14] reported a sequence-based method for prediction of metalloproteome. More recently, Passerini *et al.* proposed a new graph-based algorithm to predict the transition-metal-binding sites coordinated by Cys and His from sequence alone [15].

The increasing availability of high-quality structures deposited in PDB, makes it possible to fully explore the important structural information to accurately predict metal-binding sites, especially zinc-binding sites. In this regard, Sodhi *et al.* [16] developed a MetSite method using a variety of structural features to predict the metal-binding residues in low-resolution structural models. The Fold-X program, originally developed by Schymkowitz *et al.*, used empirical force field to predict the metal binding sites and binding affinities for the metal ions by analyzing the protein structures [17]. Goyal and Mande [18] reported a method focusing on the geometrical constraints for the prediction of zinc binding sites. The CHED method can be used to predict transition metal-binding sites from apo protein structures [4]. The FEATURE method [19], [20] applies a Bayesian classifier to recognize zinc-binding sites using a variety of physical and chemical properties that represent the microenvironment in six concentric spherical shells around the central points of zinc. Bordner developed a computational method called SitePredict that trained a Random Forest (RF) classifier using the backbone structure information to predict the metal ions and small molecules based on diverse residue properties, including spatial clustering of residue type, median-relative solvent accessible surface area and evolutionary conservation [7]. More recently, a new structure-based approach TEMSP was reported, which uses only the main chain structure of the target protein for the prediction of zinc-binding proteins [21].

Although there has been some progress for developing methods for predicting zinc-binding sites, the precision and recall of most available methods are relative low. With the rapidly increasing number of high-quality structures generated by structural genomics projects and deposited in PDB, it remains an important and pressing task to develop more accurate prediction methods that can be able to reliably identify zinc-binding or any other types of metal-binding sites, which will aid in and complement to functional annotation efforts. In particular, it is possible to develop more accurate predictors of zinc-binding sites by taking advantage of powerful machine learning algorithms, informative feature extraction and selection methods based on high-quality structural benchmark datasets. In this study, we propose an improved, generic framework based on the RF algorithm for identifying zinc-binding sites in proteins by focusing on four types of residues Cys, His, Glu and Asp (CHED) of the target proteins. Our approach takes zinc-binding residues that play catalytic or structural roles as positive samples, and the remaining as negative samples. It integrates four major types of the most important features selected by a two-step feature selection procedure, including sequence-based, structure-based, graph-theoretic network and other features into the RF classifier. We investigate to what extent each selected feature is relevant to zinc-binding site prediction by removing the feature from the feature set and examining the performance of the resulting models. We further show that this novel computational framework achieves an improved overall performance on a high-quality benchmark dataset and is capable of more accurately predicting zinc-binding residues compared to two other state-of-the-art predictors zincfinder and SitePredict.

## Methods

### Overview of our methodology

**Figure 1:**
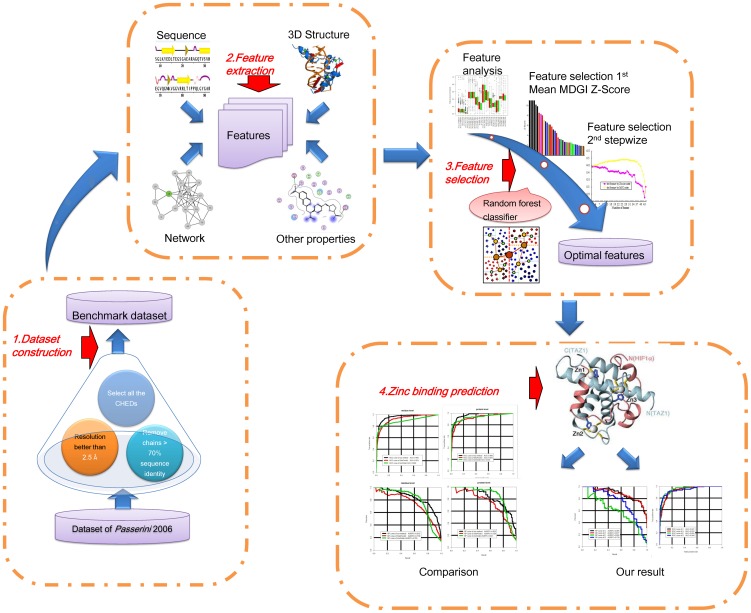
Overview of our methodology. There are four major stages: dataset construction, feature extraction, feature selection and zinc binding prediction.

An overview of our methodology is depicted in Fig. 1. As can be seen, there are four major stages: dataset construction, feature extraction, feature selection and zinc-binding prediction. At the first stage of dataset construction, all the four major types of zinc-binding residues, i.e. CHEDs are selected from the target proteins in the curated benchmark dataset. In the second stage, these CHEDs are further encoded into feature vectors which are represented based on a local window of residues centered at each selected CHED by making use of four generic categories of features: sequence, 3D structure, network and other features. In the third stage, we perform extensive feature selection to characterize the most important and contributive features that are relevant to the prediction by recursively training RF classifiers and examining their prediction accuracy. The classifiers are trained based on diverse residue- and protein-based features. In the final stage, we optimize the RF-based classifiers using the selected important features and apply the trained classifiers to predict zinc-binding residues and compare the performance of our method with the other two methods.

### Dataset

The benchmark dataset used in this study was derived from a protein dataset that was previously prepared by Passerini *et al.* [22]. This dataset contains 2,727 protein chains with zero HSSP, i.e. no pair of proteins had HSSP value>0 by running UniqueProt [23]. The HSSP value measures the pairwise sequence similarity between the two sequences by taking into account both sequence identity and sequence length [23]. We extracted proteins with a resolution better than 2.5 Å, which resulted in a final reduced dataset of 1,103 chains.

We finally selected a total of 49,631 CHEDs from the 1,103 chains. Among them, 144 chains bind to at least one zinc ion (See Table 1 for statistics), 79% of which are Zn3 or Zn4 binding chains (defined as “Znx”, where “x” denotes the number of amino acid residues bound to zinc ions). The total number of residues binding to zinc is 484. Among these residues, more than 86% of the selected Cys, His, Glu and Asp residues bind to Zn3 or Zn4. Residues are considered as binding to zinc if the distance between the atoms of the residue and zinc [24] is within a threshold of 3 Å. There are totally 223 zinc atoms binding to the selected 144 chains, as shown in Table 1.

**Table 1:** Statistics of four zinc-binding residue types (CHED) grouped based on the type of zinc ions.

	C	H	E	D	Total	Atoms	Chains
Zn1	1	10	10	9	30	30	19
Zn2	3	32	26	15	76	42	36
Zn3	17	66	24	23	130	44	35
Zn4	91	120	46	29	286	82	64
Co-catalytic Zn	22	34	10	12	68	25	14
Functional zinc	130	220	80	54	484	151	113

Znx, where “x” denotes the number of residues that bind to zinc ions. “x” = 1, 2, 3 and 4, respectively; Co-catalytic Zn: zinc ions bind to other metal ions which coordinate with side chain atoms or water molecules; Functional zinc: Zn3, Zn4 and Co-catalytic Zn ions.

Zinc ions of zinc metalloproteins are significant for their catalytic and structural functions. Zinc ions that bind to four residues (defined as “Zn4”) are considered as structural zinc, which maintain the stability of the protein but are not involved in any biochemical reaction. Zinc ions bind to three residues (defined as “Zn3”) are generally catalytic zinc, which bind to molecules and may be involved in a reaction. Except for Zn3 and Zn4, other zinc ions require cooperation with other metal ions in order to fulfill the function, usually linking with side chain atoms or water molecules. These zinc ions are called co-catalytic zinc (Co-catalytic Zn) [12]. Apart from the above zinc types, some zinc ions only bind to one or two residues; they are located on the surface of the zinc metalloproteins and lack the biological function. In this study, we only focused on the functional zinc ions (i.e. Zn3, Zn4 and Co-catalytic Zn which have catalytic or structural roles [25], [26]). In summary, CHEDs binding to these zinc ions are positive samples (484 residues, 130 C, 220 H, 80 E and 54 D) (Table 1), the remaining selected CHEDs are negative samples (49,147 residues).

We also generated an independent test set. We randomly divided the 484 positive samples into six parts, one of which was used as the independent test set while the remaining five parts were merged together and used as the benchmark dataset which was used for feature selection and 5-fold cross-validation test purposes. The negative samples were randomly selected with the negative to positive ratio of 6∶1 for both benchmark training and independent test datasets. Note that the independent test dataset was never used during the model training and feature selection process; the prediction model was trained based on the benchmark dataset and then applied to the independent test dataset for its performance evaluation.

Moreover, an apo protein structure dataset was produced according to the holo structures included in the current datasets, using the same strategy as described by Ebert and Altman [4]. More specifically, we first searched the PDB database by detecting the apo protein structures that do not contain zinc and share 95% or more sequence identity with the holo structures. We then identified the residues in the apo structures that correspond to those binding zinc in the holo form. Finally, we obtained a set of 31 apo/holo structures containing 96 zinc-binding residues. For evaluating the prediction performance on the apo protein structure set, the ratio of negative to positive was also set as 6∶1. The curated benchmark dataset, independent test dataset and apo protein dataset are available at http://protein.cau.edu.cn/zincidentifier/.

### Feature extraction

We select and extract a variety of features that describe different sequence, structure and network microenvironments which might be of importance for zinc-binding site prediction and which are used in combination as input to the RF-based classifiers. Features used in our framework comprise of four generic types: sequence, structure, network and other features (listed in Table 2). In particular, although solvent exposure features are generally considered as structural properties, this particular feature type is important for improving the prediction performance of zinc-binding residues. Also, we find several solvent exposure features useful for the prediction which were mostly understudied in previous work, which is discussed in the following sections. Therefore, we consider the solvent exposure feature as an additional feature type and would like to investigate its ability to improve upon the prediction versus other structure features.

1. **Sequence features.** A wide range of sequence features are used which make a significant improvement for the prediction. These include: (i) position-specific scoring matrices (PSSMs) from PSI-BLAST [27] using a default E-value cutoff with three iterations; (ii) native disorder predicted by DISOPRED2 [28]; (iii) protein aggregation properties generated by TANGO [29]; (iv) sequence length; (v) CHED residue percentage in the protein chain; (vi) conservation score directly derived from the PSSM. The conservation score is defined as:
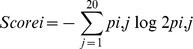
where *p_i,j_* is the frequency of amino acid *j* at position *i*. It was extracted from the PSSM generated by PSI-BLAST.
  A combination of these sequence or sequence-derived features has been shown to be able to further improve the prediction performance in our recent work [30]–[33].
2. **Structure features.** DSSP [34] is used to extract a series of structure features, including the secondary structure, solvent-accessible surface area (SASA), hydrogen bonds and protein backbone torsion angles (PHI and PSI angles). HBPLUS [35] is used to calculate the number of hydrogen bonds. NACCESS is also used to generate the respective solvent accessibility of the side chain, main chain, non-polar and polar region [36].
3. **Network features.** Graph-theoretic approach derived from the residue-residue contact graphs is becoming a useful method for unraveling the modular network organization of protein structure and predicting the protein function given the structure information [37]–[39]. If the spatial coordinate distance of the corresponding Cα atoms of two residues is within a cutoff distance threshold, the two residues are defined as being in contact. Residue-residue contact graphs were constructed using the spatial coordinates of protein structures, with a cutoff distance of 6.5 Å between the Cα atoms of two residues in the structure. We use the JUNG library (available at http://jung.sourceforge.net/) to calculate the network properties that represent the local microenvironment of residues, including clustering coefficient, degree, density, eccentricity, betweenness, closeness and status.
4. **Other features.** New solvent exposure features are also used in our study. They provide an important description of the local spatial environments of the residue of interest, providing useful information that is complementary to the traditional solvent exposure measures, such as solvent accessibility and accessible surface area [40]–[42]. We find that these features are particularly important for the prediction and thus discuss their relevance by considering them as a separate feature type from other structure features. These include the number of C_β_ atoms in the lower Half-Sphere (HSEBD), residue depth (RD), the coordination number (CN), the number of C_α_ atoms in the upper Half-Sphere (HSEAU), the number of C_β_ atoms in the upper Half-Sphere (HSEBU), the number of C_α_ atoms in the lower Half-Sphere (HSEAD) and atom depth of a residue's C_α_ atom (RDa). The hsexpo program in the Biopython package is used to calculate these solvent exposure features [43].

We use a sliding window approach with a size of 9 residues to extract all sorts of different features and take as input to train the RF models. Each residue is represented by Vn (n = 1, 2, 3, …, 9, denoting the position of the residue in such window; the centered residue is denoted as V5). Similarly, the elements in the PSSM (with a dimension of 9×20 = 180) are denoted as V1, V2, …, V180, respectively.

**Table 2:** Four generic feature types used in this study, including sequence, structure, network and other features.

Feature type	Annotation
Sequence	PSSM (PSI-BLAST [27])
	DISOPRED score [28]
	Protein aggregation properties (TANGO [29])
	Sequence length
	CHED percentage in the chain
	Conservation score
Structure	DSSP [34]
	Number of H-bonds (HBPLUS [35])
	Solvent accessibility (NACCESS [36])
	B-factor
Network	Graph-theoretic network feature
Other features	Solvent exposure (biopython [43])

### Feature selection

Due to the nature of having a large number of heterogeneous and possibly redundant features in many data mining tasks, feature selection techniques play an increasingly important role in bioinformatics applications [44]. Thus, a great deal of attention is being paid to application of these techniques that can potentially improve the prediction accuracy of machine learning classifiers, for example, in the prediction of protein folds [45], catalytic residues [46], protein crystallization [47], helix-helix interaction in membrane proteins [48] and disulfide connectivity [49]. In our study, we used a two-step feature selection method based on RF [50] to identify most informative and contributive features for the prediction of the zinc-binding sites. In the first step, the mean decrease Gini index (MDGI) was used to generate the optimal feature candidates (OFCs), which was calculated by the RF package implemented in R [51]. MDGI describes the importance of an individual vector element for correctly classifying a residue as zinc-binding or non-zinc binding. The mean MDGI Z-Score of each vector element is calculated by the following equation:
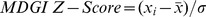
where *x_i_* is the mean MDGI of the *i*-th feature, 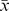 is the mean of all MDGI values of all vector elements of the feature *x*, and σ is the standard deviation (SD). We obtained the optimal feature candidates (OFCs) by selecting the features from the top 70 MDGI Z-Scores features.

The second step is a stepwise feature selection based on the RF classifier. To evaluate the prediction performance of the classifiers, 5-fold cross-validation tests were performed, where the whole benchmark dataset was randomly split into five subsets. In each validation step, one subset was left out as the testing set, while the remaining four subsets formed the reduced training set. The RF model was trained on this reduced training set and subsequently validated by testing it on the left out subset. This procedure was repeated five times, each time leaving out a different subset of the samples such that in the end each subset was in turn left out exactly once. The final performance was calculated by averaging over the five subsets. In the first round of the stepwise feature selection, we trained the RF model with the 70 optimal selected feature candidates. Then in the next round, one feature would be removed from the set of the optimal feature candidates. If the resulting RF classifier achieved a higher score (score = AUC*recall*precision, which can be considered as a balanced score incorporating the three measures AUC, recall and precision), this feature would be removed. In particular, this score was averaged over 100 times to obtain a stable value, hence the order in which features were removed in each round was almost certain. This combined score was used to evaluate the performance of the RF classifier because it is useful for comprehensively monitoring the changes of all of the three measures. The stepwise feature selection procedure continued until this score no longer increased. This procedure allows the most important and informative features to be systematically identified.

### Random forest-based classifier

In this study, we formulate the prediction task of zinc-binding sites as a binary classification problem and solve it using a machine learning approach-random forest (RF), where the zinc-binding residue is labeled with 1 and non-zinc binding residue is labeled with −1. RF is an ensemble tree-structured classifier [51]. A typical RF extends many classification trees; each tree generates a classification, and the tree “votes” for one of the two classes (positive or negative). To classify and predict whether a residue is zinc-binding or not, the physicochemical properties and the microenvironment of the selected residue are represented by a feature vector and encoded into the RF-based predictor to classify this vector as either positive (zinc-binding) or negative (non-zinc binding). The random forest classifier selects the classification as the final prediction that has the the largest number of votes (among all the trees in the forest). An advantage of RF is that it is particularly suitable for dealing with a large dataset with high-dimensional and noisy input features. In addition, as RF does not require time-consuming optimization process, the classifier training and prediction by RF are generally much faster than many other algorithms such as SVM. Due to these advantages, RF has been extensively applied in many classification and regression tasks and demonstrated satisfactory performance, for example, in predicting residue-residue contacts [52], helix-helix interaction in membrane proteins [48], DNA-binding residues [53] and protein interaction sites [54]. We use the randomForest R package [51] for the implementation of the RF algorithm.

### Performance Evaluation

We use four performance measures, namely, Recall (REC), Precision (PRE), the Area Under the Curve (AUC) and the Area Under the Recall-Precision Curve (AURPC) to evaluate the prediction performance of the methods. In our study, the dataset is heavily imbalanced with the negative to positive ratio of 100∶1. For this highly imbalanced dataset, use of the Accuracy measure, i.e. the proportion of true prediction results (true positives+true negatives) in the dataset, is not appropriate, since all-positive or all-negative classifiers can also achieve a very good classification rate. Therefore, we consider using alternative metrics such as AUC and AURPC for a comprehensive evaluation of the models' performance. AUC is the area under the receiver-operating characteristic (ROC) curve, which is a plot of true positive rate (TPR) against false positive rate (FPR). AURPC is also used in our work to evaluate the performance, which is regarded as a good alternative to AUC if there is a large skew in the class distribution [55].

The Precision is defined as:
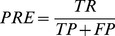
The Recall (TPR) is defined as:
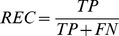
The Specificity is defined as:
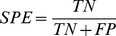
The FPR is defined as:
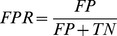
where *TP* is the number of true positives, *TN* is the number of true negatives, *FP* is the number of false positives and *FN* is the number of false negatives, respectively.

## Results and Discussion

### Two-step feature selection by random forest

By means of reducing the high dimensionality of feature vectors, feature selection can be effectively used to select the most informative features and generate a more succinct feature subset from the initial feature set with a large number of noisy and redundant features, which can allow us to significantly improve the prediction performance of machine learning-based classifiers [44]. To give insights into the feature characteristics and select the most contributive features for the prediction, we performed a two-step feature selection. The first step was to select the optimal feature candidates, while the second step was a stepwise feature selection to generate the final list of optimal features which would be used to train the best model.

**Figure 2:**
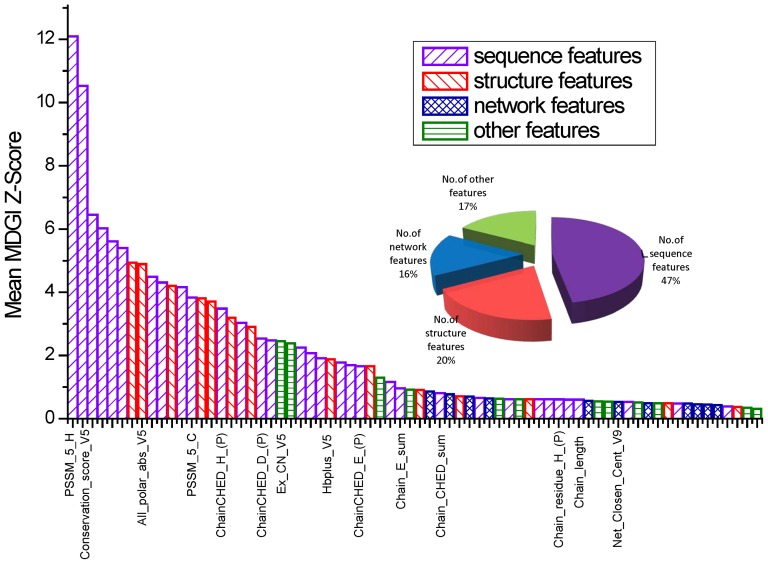
Ranking of the optimal feature candidates (OFCs) based on the MDGI Z-Scores. The bar represents the MDGI Z-Scores of the corresponding feature group. We group the features into four types: sequence, structure, network and other features, which are represented by purple, red, blue and green bars, respectively. The percentage of each of the four types of features is shown in the pie chart. The labeled features in the graph are the 14 final features selected by the two-step feature selection method.

In the first step of feature selection, we used the MDGI score to generate the initial OFCs based on a RF classifier. As a result, the top 70 features ranked were selected as the initial OFCs (Fig. 2). As can be seen, PSSM had the highest mean MDGI Z-Score, solvent accessibility feature calculated by NACCESS and solvent exposure feature also had larger Z-Scores. Meanwhile, some important features such as CHED_residue_percentage, chain length and network_closen_cent were ranked with a lower score. The percentage of each of the four types of features, as shown in the pie chart in Fig. 2, indicates that most of the contributive features in our method were predominantly derived from sequence information.

At the second step, a stepwise feature selection method was used (see the “Feature Selection” section). If the removal of a feature led to a higher prediction score of AUC*Recall*Precision, such feature would be deleted from the feature set. By iteratively removing the redundant and less informative features from the initial optimal feature candidate set, it is expected that the prediction performance could be gradually improved during this process. This two-step feature selection that combined random forest MDGI Z-Score ranking and a stepwise selection, provides a realistic approach for selecting an optimal subset of more important features within a reasonable computational time compared with other intensive feature selections [56]. In this work, this feature selection procedure assessed a total of 878 features in 9 h and 70 features in 290 h (56 rounds), respectively, on a 2*4 Xeon Intel X5650 processor Linux server (Ubuntu 10.10). Consequently, we obtained a more compact and useful feature subset that improved prediction of the RF classifiers with the application of this two-step feature selection method.

### Feature importance and contribution

**Figure 3:**
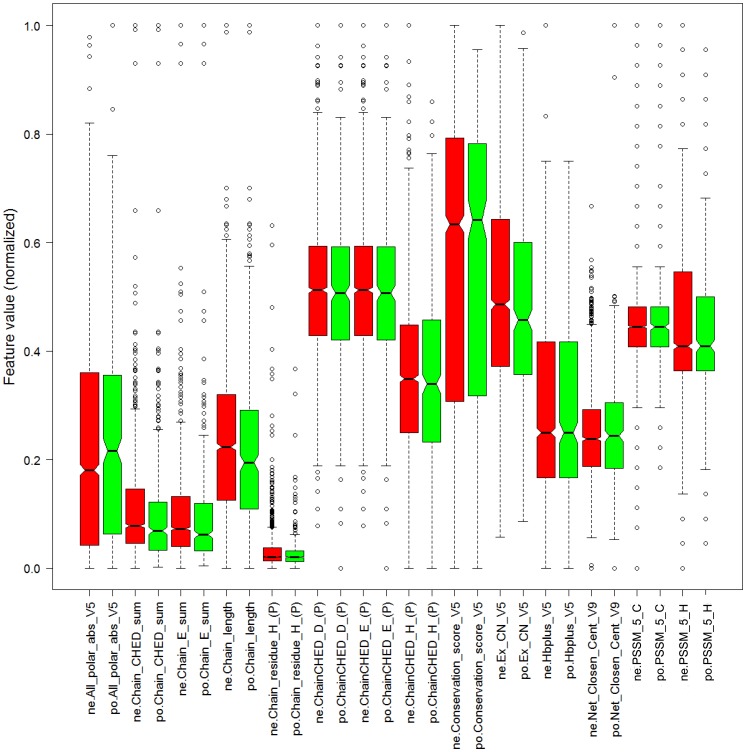
Boxplot of the 14 final features selected by a two-step feature selection that collectively made a significant contribution to zinc-binding residue prediction. Green and red boxes denote zinc-binding and non-zinc binding residues, respectively. The horizontal line in each box indicates the mean value of a corresponding feature. For each feature name in the x axis, po: positive samples (zinc-binding residues); ne: negative samples (non-binding residues).

Finally, 14 features were selected by our two-step feature selection method. The nomenclature of the selected features is specified as follows: PSSM_5_H and PSSM_5_C denote the PSSM feature in the respective positions of 9 (the “H” column in the PSSM) and 5 (the “C” column in the PSSM) of the centered residue (i.e. 5th position in the local window); Conservation_score_V5 describes the conservation score of the centered residue (Conservation feature corresponds to a 9-dimensional vector with 9 residues in the local window); All_polar_abs_V5 describes the absolute solvent accessibility of the all-polar side chain of the centered residue, which was calculated by NACCESS; ChainCHED_H_(P) denotes the percentage of His (H) in the CHEDs of the protein chain; ChainCHED_D_(P) and ChainCHED_E_(P) have similar meanings as ChainCHED_H_(P); Ex_CN_V5 and Hbplus_V5 belong to the new solvent exposure feature type and describe the coordination number (CN) and the number of H-bonds of the centered residue, respectively; Chain_E_sum and Chain_CHED_sum denote the numbers of Glu (E) and CHED in the chain, respectively; chain_length represents the number of all residues in the chain; Net_closen_cent_V9 denotes the closeness centrality of the 9th residue in the local window. In particular, inclusion of Net_closen_cent_V9 in the final feature set means that the network closeness property of neighboring residues of the centered residue in the residue-contact graph also plays an important role for prediction of zinc binding sites. The boxplots for the 14 final selected features are given in Fig. 3. It can be seen that while some feature values of the positives contrast sharply to their negative counterparts including All_polar_abs_V5, Chain_CHED_sum, Chain_E_sum, chain_length, Ex_CN_V5, other feature values did not show any obvious distinction between the positives and negatives (including chain_residue_H(P), Hbplus_V5, Net_closen_cent_V9, PSSM_5_C and PSSM_5_H), yet they collectively made a significant contribution to the performance of the final best RF model.

It is interesting to note that almost a majority of selected features were derived from sequence information rather than structure. Among the final 14 features selected by our two-step feature selection method, indeed 10 of them were derived from sequence information. In contrast, there were only two selected structure features. The possible reasons might be: (1) It has been well established that certain sequence features are important for predicting zinc-binding sites, such as the evolutionary information contained in the PSSM matrix and conservation score, which are well known features making an important contribution to the prediction performance; (2) Most initial features used in our study are derived from sequence information. For example, 70 features were selected and retained after the first step feature selection according to Z-Score, 47% of which are sequence features and most of which have relatively high Z-Scores (see Fig. 2). Therefore, it is expected that a large portion of the selected features after the second step feature selection would be derived from sequences; (3) The selected 10 sequence features have a good complementarity with other selected feature types, such as the structure and network features, thereby making a collective contribution to improved prediction of zinc-binding sites.

We further went on to elucidate the importance and contribution of the 14 final selected features at both residue and protein levels. We examined the influence of each of the 14 features on the prediction performance by iteratively removing each from the final feature set and consequently calculating the corresponding REC, PRE, AUC and AURPC of the resultant RF classifiers. The results are presented in Table 3. PSSMs and conservation score have been found useful for the prediction in previous studies [12]. In this study, they also made a great contribution to the prediction performance, especially the PSSM_5_C feature. The importance of this feature is reflected by the fact that there would be a large decrease in 7 out of 8 performance measures when we used the rest of 13 features to train the RF model. In the absence of the PSSM_5_C feature, the performance of the trained models considerably deteriorated, as reflected by the REC, AUC and AURPC at both residue and protein levels (at residue level: REC decreased from 0.804 to 0.760, AUC from 0.960 to 0.954 and AURPC from 0.832 to 0.815; at protein level: REC decreased from 0.748 to 0.703, AUC from 0.966 to 0.960, AURPC from 0.825 to 0.808). These correspond to the first and second largest performance decrease, which indicates the importance of the inclusion of this feature for zinc-binding site prediction of our method.

**Table 3:** Influence of the removal of 14 individual optimal features on the prediction performance, as evaluated by REC, PRE, AUC and AURPC.

	MDGI Z-Score	Residue level				Protein level			
		REC	PRE	AUC	AURPC	REC	PRE	AUC	AURPC
All 14 features		0.804	0.750	0.960	0.832	0.748	0.750	0.966	0.825
PSSM_5_H	12.098	0.754	0.733	0.955	0.817	0.748	0.769	0.955	0.817
Conservation_score_V5	10.527	0.769	0.745	0.956	0.828	0.775	0.748	0.965	0.831
All_polar_abs_V5	4.892	0.792	0.715	0.959	0.829	0.766	0.744	0.964	0.832
PSSM_5_C	3.836	0.760	0.748	0.954	0.815	0.703	0.709	0.960	0.808
ChainCHED_H_(P)	3.481	0.782	0.739	0.957	0.827	0.730	0.730	0.966	0.830
ChainCHED_D_(P)	2.533	0.789	0.738	0.959	0.822	0.739	0.683	0.964	0.812
Ex_CN_V5	2.451	0.806	0.732	0.958	0.824	0.730	0.730	0.965	0.814
Hbplus_V5	1.882	0.792	0.714	0.957	0.826	0.766	0.746	0.963	0.820
ChainCHED_E_(P)	1.661	0.799	0.740	0.958	0.820	0.748	0.728	0.964	0.806
Chain_E_sum	0.959	0.801	0.744	0.958	0.826	0.703	0.716	0.964	0.819
Chain_CHED_sum	0.812	0.804	0.750	0.958	0.828	0.739	0.732	0.966	0.823
Chain_residue_H_(P)	0.611	0.794	0.744	0.958	0.828	0.748	0.741	0.966	0.824
Chain_length	0.602	0.794	0.741	0.958	0.825	0.766	0.726	0.965	0.820
Net_Closen_Cent_V9	0.537	0.803	0.745	0.960	0.830	0.766	0.702	0.965	0.831

Moreover, the percentage of Glu (E) and Asp (D) in CHEDs of the protein chain also made a considerable contribution to the performance improvement, which is reflected by the changes of AURPC at both residue and protein levels by comparing the resulting performances in the presence and absence of the given features. About 96% of zinc binding residues were CHEDs in our dataset. Compared to Glu and Asp, Cys and His had higher percentages of residues that bind zinc. The percentage of Glu and Asp were only 16.5% and 11.2% in the positive CHEDs, whereas in the negative CHEDs, 41.2% and 35.7% were Glu and Asp, respectively. In other words, a protein chain is more likely to bind zinc ions if it has a lower percentage of EDs and a higher percentage of CHs. Accordingly, it is expected that the percentage of Glu and Asp in CHEDs of the chain is a useful feature for zinc-binding site prediction.

As can be seen from Table 3, there are two structure features selected in the final feature set. They are All_polar_abs_V5 (calculated by NACCESS) and Hbplus_V5 (calculated by HBPLUS), which define the absolute solvent accessibility of the all-polar side chain and the number of hydrogen bonds of the centered residue, respectively. After removal of the two features from the feature set, PRE of the resulting RF classifiers decreased from 0.750 to 0.714 in the case of Hbplus_V5 and to 0.715 in the case of All_polar_abs_V5, respectively. These correspond to the first and second largest decrease of PRE, demonstrating the importance of these two features for the prediction. In addition, there are two other features Ex_CN_V5 and Net_Closen_CentV9 in the final feature set. The former is a descriptor of solvent exposure of the centered residue in terms of the residue coordination number (CN), while the latter is a network feature which describes the mean geodesic distance from the centered residue to the neighboring node within the residue contact graph of the protein. We can see from Table 3 that Ex_CN_V5 makes a good contribution to AURPC and PRE at both residue and protein levels. However, there is not obvious decrease after the removal of the selected network feature Net_closen_cent_V9. It is well known that closeness centrality indicates a degree of residue centrality in the protein 3D structure. This feature has been previously found useful for predicting functionally important residues, including enzyme catalytic sites which are generally centrally placed in the structure [57]. However, in this study, we find that closeness centrality is not an informative feature for predicting zinc-binding sites. As zinc ions in the zinc-binding proteins are often located at the side of the structure and play structural roles (for example, zinc in zinc finger proteins helps stabilize and coordinate the alpha-helix and two beta-strands necessary for the stabilization of the structure and interaction with DNA, RNA and other molecules), the closeness centrality is not an effective feature for predicting zinc-binding sites. Yet, this feature may be useful for balancing the performance and contribute to the prediction when used in combination with other important features, which is the case when classifying the positives at other thresholds (data not shown).

### Comparison with other methods

**Figure 4:**
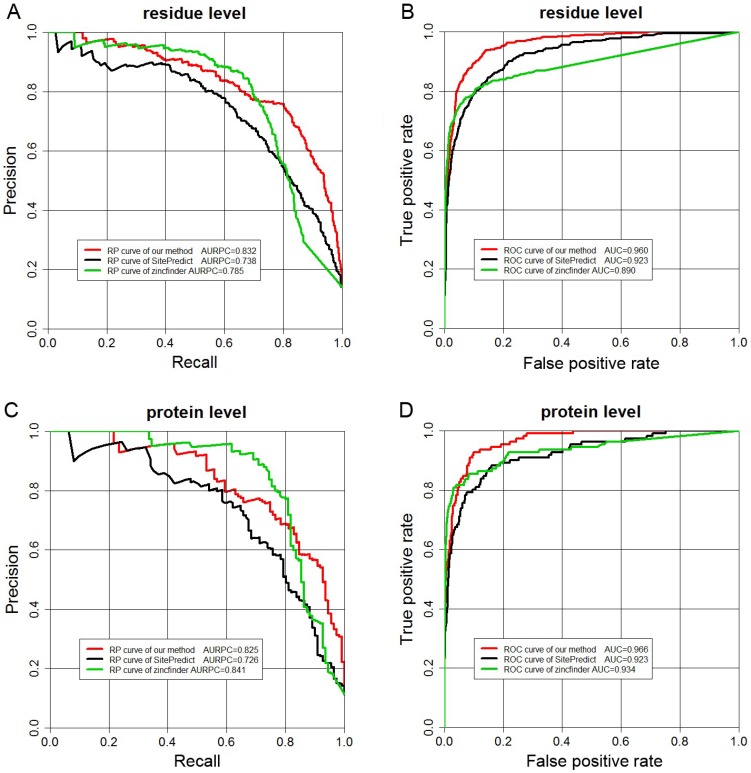
Recall-Precision and ROC curves displaying the performance of the three methods at both residue (A and B) and protein levels (C and D): our method (red line), SitePredict (black line) and zincfinder (green line) when applied to the 5-fold cross-validation benchmark dataset in this study.

We further compared our method with other previously developed methods. The performance of each of the compared methods was summarized using the AURPC, AUC, REC and PRE measures at both residue and protein levels (A true positive at the residue level was defined as a zinc-binding residue that was correctly predicted, while a true positive at the protein level was defined as a zinc-binding protein for which at least one zinc-binding residue was correctly predicted). In particular, for the sake of the comparison, we compared different methods at the fixed PRE. In addition we plotted the ROC and Recall-Precision curves to show the performance of each method (Fig. 4). ROC curves were plotted using the “ROCR” package [58] at the two levels. Recall-Precision curve is a measure of the overall quality of the prediction and is often used as an alternative complement to ROC curves especially when analyzing datasets with a large imbalance in the class distribution [59] by incorporating both the Recall and Precision measures. The AURPC was calculated using the method of Davis and Goadrich [55]. Although most zinc-binding site prediction studies predominantly evaluate the performance using AURPC, we employed both AUC and AURPC to comprehensively evaluate the performance of different methods in this study.

There exist several methods that have demonstrated good performance for predicting zinc-binding residues [7], [11], [12]. However, due to the unavailability of webserver or standalone software, some methods could not be directly compared to our method. Moreover certain methods can only be applied to predict zinc-binding proteins [21] and are not capable of predicting zinc-binding residues, while others predict the spatial position of zinc-binding or metal-binding sites [19] rather than zinc-binding residues. In addition, as our benchmark dataset was derived from the previous dataset on which Shu *et al.*'s method was built, we cannot compare with that method either. Therefore, we compared the predictive performance of our method with another two state-of-the-art methods that focused on prediction of zinc-binding residues, namely, SitePredict [7] and zincfinder [11]. The prediction performance of the three methods is shown in Table 4.

**Table 4:** Performance comparison between our method, zincfinder and SitePredict on the 5-fold cross-validation benchmark dataset.

Method	Residue level					Protein level				
	REC	PRE	SPE	AUC	AURPC	REC	PRE	SPE	AUC	AURPC
zincfinder	0.728	0.750	0.961	0.890	0.785	0.801	0.750	0.966	0.934	0.841
SitePredict	0.625	0.750	0.966	0.923	0.738	0.649	0.750	0.973	0.923	0.726
Our method	0.804	0.750	0.957	0.960	0.832	0.748	0.750	0.968	0.966	0.825
Sequence^a^	0.752	0.750	0.959	0.956	0.825	0.733	0.750	0.969	0.963	0.821

The performance was evaluated using REC, PRE, SPE, AUC and AURPC measures.

a Prediction performance of the RF model using the 10 selected sequence features only.

SitePredict is a structure-based method that also uses the RF algorithm to predict zinc-binding sites [7]. The RF classifiers of SitePredict were trained on diverse residue-based site properties comprising of spatial clustering of residue types and evolutionary conservation. We tested the performance of SitePredict by submitting our 5-fold cross-validation (benchmark) dataset to its online webserver. At the residue level, SitePredict generated 252 TP, 84 FP and 151 FN predictions, which corresponds to a REC of 0.625 at the fixed PRE of 0.750. In contrast, our method produced 324, 108 and 79 for TP, FP and FN predictions in the 5-fold cross-validation tests, corresponding to a REC of 0.804 and a PRE of 0.750, respectively. Although our method predicted more FPs than SitePredict, the REC of our method is significantly better than that of SitePredict (0.804 versus 0.625), which means that our method is more accurate in predicting the positive samples (zinc-binding residues) than SitePredict. Meanwhile, AUC was also improved from 0.923 to 0.960, implying that our method outperformed SitePredict. We also compared our method with SitePredict using AURPC. From Fig. 4A, we can see that the Recall-Precision curve of our method is consistently higher than that of SitePredict, with AURPC increased from 0.738 to 0.832. At the protein level, we also observe a similar tendency that the performance of our method is better than that of SitePredict, with REC, AUC and AURPC increased from 0.649, 0.923 and 0.726 to 0.748, 0.966 and 0.825, respectively, suggesting that our method provides a better classification performance for recognizing zinc-binding sites than SitePredict.

Zincfinder is a sequence-based tool for zinc-binding site prediction using SVMs [11]. Zincfinder was applied to our benchmark set and generated 288 TP, 96 FP and 108 FN predictions, respectively at the residue level, with a REC of 0.728 at the equal PRE of 0.750. Compared to zincfinder, REC of our method was increased to 0.804. Zincfinder achieved AUC and AURPC which were respectively worse than those of our method at the residue level, but it attained a higher AURPC at the protein level (0.825 versus 0.841, Table 4). While our method was outperformed by zincfinder at the protein level (reflected by the REC and AURPC), it achieved a predictive power comparable to, if not better than, zincfinder with lower REC and AURPC at the protein level, but with higher REC and AURPC at the residue level as well as higher AUC at both levels.

**Figure 5:**
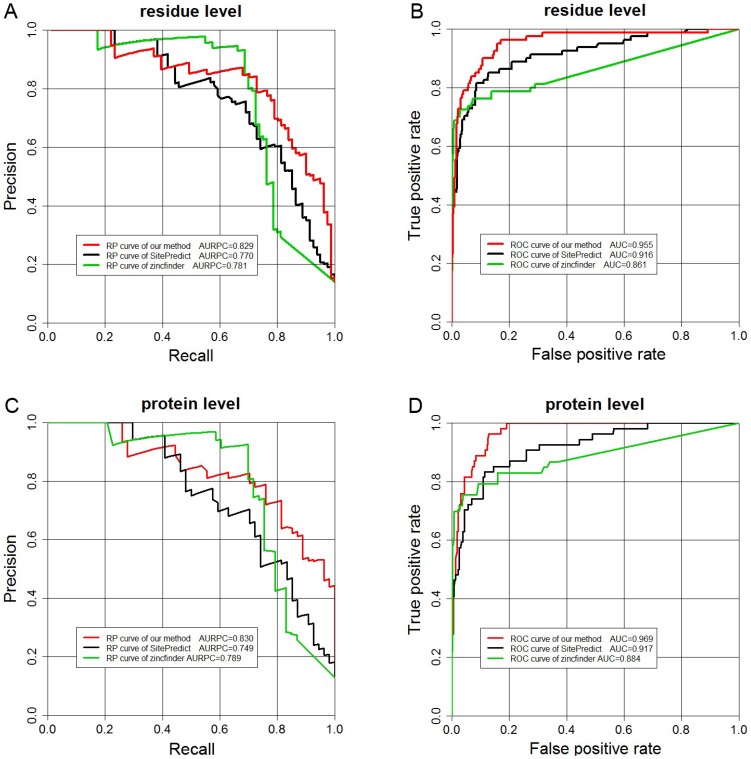
Recall-Precision and ROC curves displaying the performance of the three methods at both residue (A and B) and protein levels (C and D): our method (red line), SitePredict (black line) and zincfinder (green line) when applied to the independent test set in this study.

We also compared our method with these two methods on the independent test set. The performance is shown in Table 5. We can see that the REC, AURPC and AUC of our method are respectively better than those of the other two methods at both residue and protein levels. In addition we plotted the Recall-Precision and ROC curves at the two levels in Fig. 5. The Recall-Precision and ROC curves have a similar tendency as those on the benchmark set at the two levels. Overall, these results suggest that our method has outperformed SitePredict and zincfinder on this independent test set.

**Table 5:** Performance comparison between our method, zincfinder and SitePredict on the independent test dataset.

Method	Residue level					Protein level				
	REC	PRE	SPE	AUC	AURPC	REC	PRE	SPE	AUC	AURPC
Zincfinder	0.725	0.750	0.961	0.861	0.781	0.717	0.750	0.966	0.884	0.789
SitePredict	0.691	0.750	0.976	0.916	0.770	0.574	0.750	0.972	0.917	0.749
Our method	0.790	0.750	0.958	0.955	0.829	0.759	0.750	0.964	0.969	0.830
Sequence	0.753	0.750	0.960	0.934	0.823	0.763	0750	0.961	0.950	0.826
Apo^a^	0.719	0.750	0.958	0.953	0.713	0.742	0.750	0.973	0.970	0.762

The performance was evaluated using REC, PRE, SPE, AUC and AURPC measures.

a Prediction performance of the RF model on the apo protein structure dataset containing 31 apo structures.

Considering that zincfinder used only sequence features and our method also had a majority of selected features derived from sequence information, we also trained our model using the 10 selected sequence features only, applied it to make the prediction, and compared the results with zincfinder (See Table 4 and Table 5, respectively). It can be seen that our method is able to provide a competitive and in some cases better performance than zincfinder in terms of Recall, AUC and AURPC at both residue and protein levels based on the benchmark and independent datasets. This suggests that our method that only used the sequence features has also outperformed zincfinder. Overall, the results indicate that zincfinder has a better ability to predict more TPs at the protein level while our method is more accurate and displays a greater predictive power at the residue level. In addition, it is noteworthy that there is an overlap of more than 60% of protein chains in our training set and the benchmark dataset of zincfinder, which may explain why the sequence-based tool zincfinder was better than the other structure-based tool SitePredict and why our method was outperformed by zincfinder at the protein level. Altogether, our method provides a competitive performance compared to the other two state-of-the-art methods on the 5-fold cross-validation benchmark dataset.

### Predictions for the apo protein structures

Next, we examined how well our method performs on the apo protein structures, the prediction of which is more relevant for the actual use of our approach. We thus evaluated the performance of our approach based on the generated apo protein structure dataset (See Dataset section). The performance is shown in Table 5. As can be seen, our method achieved the Recall of 0.719 and 0.742 at a corresponding Precision of 0.750 at the respective residue and protein levels on this apo structure dataset. The AUC is 0.953 and 0.970, AURPC is 0.713 and 0.762 at the two levels. Despite the limited size of the apo structure dataset, these results indicates that our method is less sensitive to the structural rearrangements due to the binding of zinc ion, and that our method can be used to identify the zinc-binding sites in the apo protein structures.

### Predictions for the four types of zinc-binding residues

**Figure 6:**
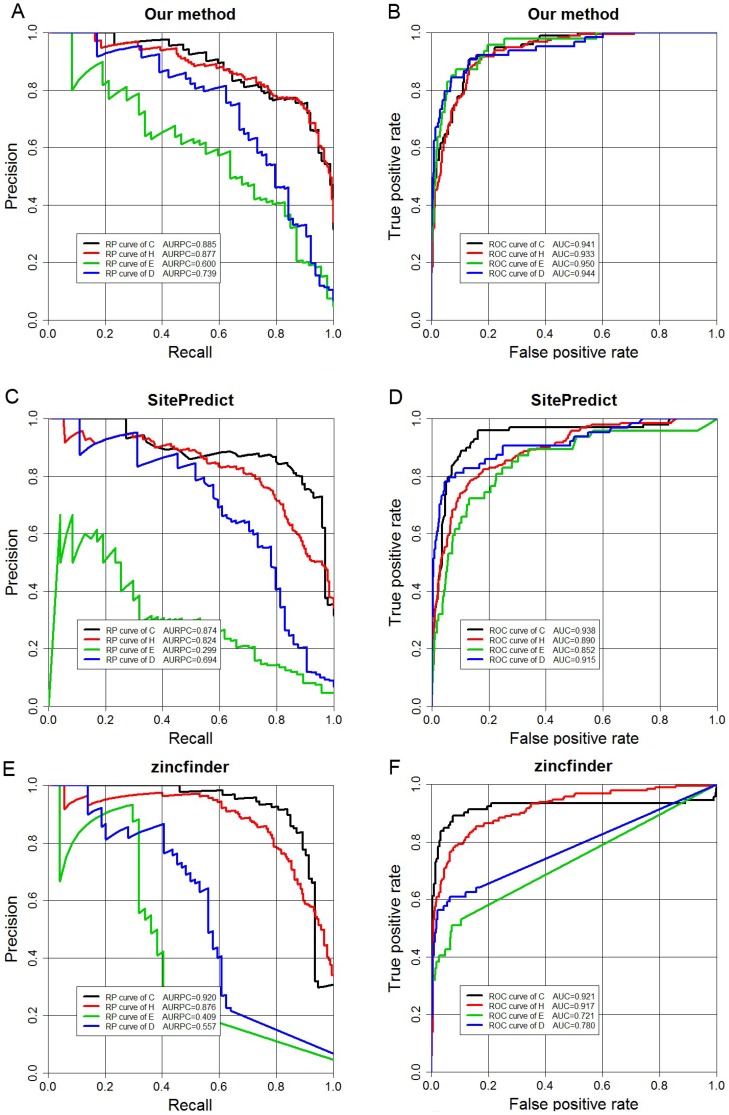
Recall-Precision and ROC curves displaying the performance of the three methods for predicting four types of zinc-binding residues CHED when applied to the 5-fold cross-validation benchmark dataset. (A) and (B): Recall-Precision and ROC curves for our method; (**C**) and (**D**): Recall-Precision and ROC curves for SitePredict; (**E**) and (**F**): Recall-Precision and ROC curves for zincfinder, respectively. Cys (C), black line; His (H), red line; E (Glu), green line; D (Asp), blue line.

As discussed above, there are four major types of residues that bind to zinc- Cys, His, Glu and Asp (CHED). Table 6 shows a comparison of the predictive performances of the three different tools for predicting these types on the training set. Cys and His were predicted with higher accuracy than Glu and Asp. For example, the REC values of Cys and His are 0.909 and 0.891 at an equal PRE of 0.750, respectively, which are much higher than those of Glu and Asp, which only had REC values of 0.319 and 0.656, respectively. A similar tendency was observed for AURPC. On the other hand, we note that AUCs of Glu and Asp are higher than those of Cys and His. This is mainly because Cys and His have a higher percentage in positive samples (zinc-binding residues) while Glu and Asp have a higher percentage in negative samples (non-binding residues). The proportions of zinc binding residues of CHED are C: 26.9%, H: 45.5%, E: 16.5% and D: 11.2%. In contrast, the proportions of all CHED samples are C: 10.9%, H: 19.7%, E: 36.1% and D: 33.3%. Hence, the larger percentage of Glu and Asp in negative examples resulted in much more FP predictions than those of Cys and His, leading to a lower PRE. It is also why AURPC was decreased (Fig. 6A) with the decrease of REC. REC indicates the prediction performance of positive samples, which does not necessarily reflect the predictive performance of negative samples. From this perspective, AUC can be considered as a balanced measure of the overall quality of the prediction as it incorporates both SEN (the same as REC) and SPE (which is TN/(TN+FP)) measures. A higher ROC curve of Glu and Asp indicates a better accuracy of negative samples (Fig. 6B). Therefore, AUC is also an important performance measure of our method, playing a crucial rule that is complementary to AURPC. For the sake of comparison, we also list the predictive performance of CHED by another two methods SitePredict and zincfinder in Table 6. We can see that there are straight lines in some of the precision-recall and ROC curves for zincfinder, for example, the ROC curves of ASP, this is mainly because that for 83.3% of negative samples of Asp (D) residues, the predicted value of zincfinder is 0. Similarly, 89.7% of negative samples of Glu (E) residues were predicted as 0, thus there were no data for zincfinder with FPR>0.103 and as a result there was a straight line in its ROC curve.

**Table 6:** Predictive performance of the four types of zinc-binding residues (CHED) of our method, SitePredict and zincfinder on the 5-fold cross-validation benchmark dataset.

Method	Residue type	REC	PRE	AUC	AURPC
Our method	C	0.909	0.750	0.941	0.885
	H	0.891	0.750	0.933	0.877
	E	0.319	0.750	0.950	0.600
	D	0.656	0.750	0.944	0.739
SitePredict	C	0.919	0.750	0.938	0.874
	H	0.782	0.750	0.890	0.824
	E	0.100	0.750	0.852	0.299
	D	0.578	0.750	0.915	0.694
zincfinder	C	0.914	0.750	0.921	0.920
	H	0.833	0.750	0.917	0.876
	E	0.319	0.750	0.721	0.409
	D	0.453	0.750	0.780	0.557

## Conclusion

We have developed a powerful computational framework that combines a variety of sequence, structure, graph-theoretic network and other features of residues to improve prediction of zinc-binding sites, which relies on a two-step RF algorithm to retrieve and select the most useful and contributive features to train the prediction models. Using this integrative framework, we investigated what information can be retrieved from the different types of residue microenvironments that is relevant to the prediction of zinc-binding sites. By focusing on four major types of residues: Cys, His, Glu and Asp, it achieved 80% recall at 75% precision in 5-fold cross-validation tests on the benchmark dataset containing 1,103 high-quality structures and 484 zinc-binding residues. We found that Cys and His were generally better predicted with respective recalls of 91% and 89%, which were much higher than those of Glu and Asp. We also performed independent test and the results showed that our method provided a higher REC, AUC and AURPC than SitePredict and zincfinder at both residue and protein levels. Benchmarking experiments showed that our method has outperformed the two other state-of-the-art methods at the corresponding 75% precision on both benchmark and independent test datasets. In summary, the performance improvement of our method may be attributed to three important ingredients: (i) construction of a high-quality non-redundant structural benchmark dataset; (ii) integration of a variety of features including sequence, structure, graph-theoretic network and other features and inclusion of novel features that collectively make a good contribution to the performance; (iii) a two-step feature selection method to remove the overlapping and redundant features. In addition, this framework is generally applicable to many other classification problems in structural bioinformatics and can be readily extended to solve the prediction task of other types of metal-binding or ligand-binding sites. We believe that it can be an effective tool for accurately identifying zinc-binding sites with the increasing availability of high-quality structure data.

## References

[1] Holm RH, Kennepohl P, Solomon EI (1996) Structural and Functional Aspects of Metal Sites in Biology. Chem Rev 96: 2239–2314. 11848828 10.1021/cr9500390

[2] Matthews JM, Loughlin FE, Mackay JP (2008) Designed metal-binding sites in biomolecular and bioinorganic interactions. Curr Opin Struct Biol 18: 484–490. 18554898 10.1016/j.sbi.2008.04.009

[3] Bernstein FC, Koetzle TF, Williams GJ, Meyer EF, Brice MD, et al (1977) The Protein Data Bank: a computer-based archival file for macromolecular structures. J Mol Biol 112: 535–542. 875032 10.1016/s0022-2836(77)80200-3

[4] Babor M, Gerzon S, Raveh B, Sobolev V, Edelman M (2008) Prediction of transition metal-binding sites from apo protein structures. Proteins 70: 208–217. 17657805 10.1002/prot.21587

[5] Coleman JE (1992) Zinc proteins: enzymes, storage proteins, transcription factors, and replication proteins. Annu Rev Biochem 61: 897–946. 1497326 10.1146/annurev.bi.61.070192.004341

[6] Andreini C, Banci L, Bertini I, Rosato A (2006) Counting the zinc-proteins encoded in the human genome. J Proteome Res 5: 196–201. 16396512 10.1021/pr050361j

[7] Bordner AJ (2008) Predicting small ligand binding sites in proteins using backbone structure. Bioinformatics 24: 2865–2871. 18940825 10.1093/bioinformatics/btn543 PMC2639300

[8] Lin CT, Lin KL, Yang CH, Chung IF, Huang CD, et al (2005) Protein metal binding residue prediction based on neural networks. Int J Neural Syst 15: 71–84. 15912584 10.1142/S0129065705000116

[9] Lin HH, Han LY, Zhang HL, Zheng CJ, Xie B, et al (2006) Prediction of the functional class of metal-binding proteins from sequence derived physicochemical properties by support vector machine approach. BMC Bioinformatics 7: S13. 10.1186/1471-2105-7-S5-S13 PMC1764469 17254297

[10] Menchetti S, Passerini A, Frasconi P, Andreini C, Rosato A (2006) Improving prediction of zinc binding sites by modeling the linkage between residues close in sequence. Research in Computational Molecular Biology, Proceedings 3909: 309–320.

[11] Passerini A, Andreini C, Menchetti S, Rosato A, Frasconi P (2007) Predicting zinc binding at the proteome level. BMC Bioinformatics 8: 39. 17280606 10.1186/1471-2105-8-39 PMC1800866

[12] Shu N, Zhou TP, Hovmoller S (2008) Prediction of zinc-binding sites in proteins from sequence. Bioinformatics 24: 775–782. 18245129 10.1093/bioinformatics/btm618

[13] Lippi M, Passerini A, Punta M, Rost B, Frasconi P (2008) MetalDetector: a web server for predicting metal-binding sites and disulfide bridges in proteins from sequence. Bioinformatics 24: 2094–2095. 18635571 10.1093/bioinformatics/btn371 PMC2732205

[14] Andreini C, Bertini I, Rosato A (2009) Metalloproteomes: a bioinformatic approach. Acc Chem Res 42: 1471–1479. 19697929 10.1021/ar900015x

[15] Passerini A, Lippi M, Frasconi P (2012) Predicting Metal-Binding Sites from Protein Sequence. Ieee-Acm Transactions on Computational Biology and Bioinformatics 9: 203–213. 21606549 10.1109/TCBB.2011.94

[16] Sodhi JS, Bryson K, McGuffin LJ, Ward JJ, Wernisch L, et al (2004) Predicting metal-binding site residues in low-resolution structural models. J Mol Biol 342: 307–320. 15313626 10.1016/j.jmb.2004.07.019

[17] Schymkowitz JWH, Rousseau F, Martins IC, Stricher F, Serrano L, et al (2005) Prediction of water and metal binding sites and their affinities by using the Fold-X force field. Proc Natl Acad Sci U S A 102: 10147–10152. 16006526 10.1073/pnas.0501980102 PMC1177371

[18] Goyal K, Mande SC (2008) Exploiting 3D structural templates for detection of metal-binding sites in protein structures. Proteins-Structure Function and Bioinformatics 70: 1206–1218. 10.1002/prot.21601 17847089

[19] Ebert JC, Altman RB (2008) Robust recognition of zinc binding sites in proteins. Protein Science 17: 54–65. 18042678 10.1110/ps.073138508 PMC2144590

[20] Wu S, Liu T, Altman RB (2010) Identification of recurring protein structure microenvironments and discovery of novel functional sites around CYS residues. BMC Struct Biol 10: 4. 20122268 10.1186/1472-6807-10-4 PMC2833161

[21] Zhao W, Xu M, Liang Z, Ding B, Niu LW, et al (2011) Structure-based de novo prediction of zinc-binding sites in proteins of unknown function. Bioinformatics 27: 1262–1268. 21414989 10.1093/bioinformatics/btr133

[22] Passerini A, Punta M, Ceroni A, Rost B, Frasconi P (2006) Identifying cysteines and histidines in transition-metal-binding sites using support vector machines and neural networks. Proteins-Structure Function and Bioinformatics 65: 305–316. 10.1002/prot.21135 16927295

[23] Mika S, Rost B (2003) UniqueProt: creating representative protein sequence sets. Nucleic Acids Research 31: 3789–3791. 12824419 10.1093/nar/gkg620 PMC169026

[24] Harding MM (2004) The architecture of metal coordination groups in proteins. Acta Crystallographica Section D-Biological Crystallography 60: 849–859. 10.1107/S0907444904004081 15103130

[25] Vallee BL, Auld DS (1992) Functional Zinc-Binding Motifs in Enzymes and DNA-Binding Proteins. Faraday Discussions 93: 47–65. 10.1039/fd9929300047 1290939

[26] Auld DS (2001) Zinc coordination sphere in biochemical zinc sites. Biometals 14: 271–313. 11831461 10.1023/a:1012976615056

[27] Altschul SF, Madden TL, Schaffer AA, Zhang JH, Zhang Z, et al (1997) Gapped BLAST and PSI-BLAST: a new generation of protein database search programs. Nucleic Acids Research 25: 3389–3402. 9254694 10.1093/nar/25.17.3389 PMC146917

[28] Ward JJ, Sodhi JS, McGuffin LJ, Buxton BF, Jones DT (2004) Prediction and functional analysis of native disorder in proteins from the three kingdoms of life. J Mol Biol 337: 635–645. 15019783 10.1016/j.jmb.2004.02.002

[29] Fernandez-Escamilla AM, Rousseau F, Schymkowitz J, Serrano L (2004) Prediction of sequence-dependent and mutational effects on the aggregation of peptides and proteins. Nature Biotechnology 22: 1302–1306. 10.1038/nbt1012 15361882

[30] Song J, Yuan Z, Tan H, Huber T, Burrage K (2007) Predicting disulfide connectivity from protein sequence using multiple sequence feature vectors and secondary structure. Bioinformatics 23: 3147–3154. 17942444 10.1093/bioinformatics/btm505

[31] Song J, Tan H, Shen HB, Mahmood K, Boyd SE, et al (2010) Cascleave: towards more accurate prediction of caspase substrate cleavage sites. Bioinformatics 26: 752–760. 20130033 10.1093/bioinformatics/btq043

[32] Song J, Tan H, Wang M, Webb GI, Akutsu T (2012) TANGLE: two-level support vector regression approach for protein backbone torsion angle prediction from primary sequences. PLoS One 7: e30361. 22319565 10.1371/journal.pone.0030361 PMC3271071

[33] Wang M, Zhao XM, Takemoto K, Xu H, Li Y, et al (2012) FunSAV: predicting the functional effect of single amino acid variants using a two-stage random forest model. PLoS One e43847. 22937107 10.1371/journal.pone.0043847 PMC3427247

[34] Kabsch W, Sander C (1983) Dictionary of Protein Secondary Structure - Pattern-Recognition of Hydrogen-Bonded and Geometrical Features. Biopolymers 22: 2577–2637. 6667333 10.1002/bip.360221211

[35] Mcdonald IK, Thornton JM (1994) Satisfying Hydrogen-Bonding Potential in Proteins. J Mol Biol 238: 777–793. 8182748 10.1006/jmbi.1994.1334

[36] Hubbard SJ, Thornton JM (1993) ‘NACCESS’. Computer Program: Department Biochemistry and Molecular Biology, University College, London.

[37] Li Y, Li G, Wen Z, Yin H, Hu M, et al (2011) Novel feature for catalytic protein residues reflecting interactions with other residues. PLoS One 6: e16932. 21468322 10.1371/journal.pone.0016932 PMC3066176

[38] Li Y, Wen Z, Xiao J, Yin H, Yu L, et al (2011) Predicting disease-associated substitution of a single amino acid by analyzing residue interactions. BMC Bioinformatics 12: 14. 21223604 10.1186/1471-2105-12-14 PMC3027113

[39] Maetschke SR, Yuan Z (2009) Exploiting structural and topological information to improve prediction of RNA-protein binding sites. BMC Bioinformatics 10: 341. 19835626 10.1186/1471-2105-10-341 PMC2774325

[40] Chakravarty S, Varadarajan R (1999) Residue depth: a novel parameter for the analysis of protein structure and stability. Structure 7: 723–732. 10425675 10.1016/s0969-2126(99)80097-5

[41] Song J, Tan H, Takemoto K, Akutsu T (2008) HSEpred: predict half-sphere exposure from protein sequences. Bioinformatics 24: 1489–1497. 18467349 10.1093/bioinformatics/btn222

[42] Song J, Tan H, Mahmood K, Law RH, Buckle AM, et al (2009) Prodepth: predict residue depth by support vector regression approach from protein sequences only. PLoS One 4: e7072. 19759917 10.1371/journal.pone.0007072 PMC2742725

[43] Hamelryck T (2005) An amino acid has two sides: A new 2D measure provides a different view of solvent exposure. Proteins 59: 38–48. 15688434 10.1002/prot.20379

[44] Saeys Y, Inza I, Larranaga P (2007) A review of feature selection techniques in bioinformatics. Bioinformatics 23: 2507–2517. 17720704 10.1093/bioinformatics/btm344

[45] Chen K, Kurgan L (2007) PFRES: protein fold classification by using evolutionary information and predicted secondary structure. Bioinformatics 23: 2843–2850. 17942446 10.1093/bioinformatics/btm475

[46] Zhang T, Zhang H, Chen K, Shen S, Ruan J, et al (2008) Accurate sequence-based prediction of catalytic residues. Bioinformatics 24: 2329–2338. 18710875 10.1093/bioinformatics/btn433

[47] Mizianty MJ, Kurgan L (2011) Sequence-based prediction of protein crystallization, purification and production propensity. Bioinformatics 27: i24–i33. 21685077 10.1093/bioinformatics/btr229 PMC3117383

[48] Wang XF, Chen Z, Wang C, Yan RX, Zhang ZD, et al (2011) Predicting Residue-Residue Contacts and Helix-Helix Interactions in Transmembrane Proteins Using an Integrative Feature-Based Random Forest Approach. PLoS One 6: e26767. 22046350 10.1371/journal.pone.0026767 PMC3203928

[49] Zhu L, Yang J, Song J, Chou KC, Shen HB (2010) Improving the accuracy of predicting disulfide connectivity by feature selection. J Comput Chem 31: 1478–1485. 20127740 10.1002/jcc.21433

[50] Breiman L (2001) Random forests. Machine Learning 45: 5–32.

[51] Liaw A, Watthew M (2002) Classification and Regression by randomForest. R news 2: 18–22.

[52] Li YQ, Fang YP, Fang JW (2011) Predicting residue-residue contacts using random forest models. Bioinformatics 27: 3379–3384. 22016406 10.1093/bioinformatics/btr579

[53] Wu JS, Liu HD, Duan XY, Ding Y, Wu HT, et al (2009) Prediction of DNA-binding residues in proteins from amino acid sequences using a random forest model with a hybrid feature. Bioinformatics 25: 30–35. 19008251 10.1093/bioinformatics/btn583 PMC2638931

[54] Chen XW, Jeong JC (2009) Sequence-based prediction of protein interaction sites with an integrative method. Bioinformatics 25: 585–591. 19153136 10.1093/bioinformatics/btp039

[55] Davis J, Goadrich M (2006) The relationship between Precision-Recall and ROC Curves. In Proceedings of the 23rd international conference on Machine learning ACM Press, Pittsburgh, Pennsylvania.

[56] Ebina T, Toh H, Kuroda Y (2011) DROP: an SVM domain linker predictor trained with optimal features selected by random forest. Bioinformatics 27: 487–494. 21169376 10.1093/bioinformatics/btq700

[57] Chea E, Livesay DR (2007) How accurate and statistically robust are catalytic site predictions based on closeness centrality? BMC Bioinformatics 8: 153. 17498304 10.1186/1471-2105-8-153 PMC1876251

[58] Sing T, Sander O, Beerenwinkel N, Lengauer T (2005) ROCR: visualizing classifier performance in R. Bioinformatics 21: 3940–3941. 16096348 10.1093/bioinformatics/bti623

[59] Zhang JP, Bloedorn E, Rosen L, Venese D (2004) Learning rules from highly unbalanced data sets. Fourth Ieee International Conference on Data Mining, Proceedings 571–574.

